# Medicine and the Law

**Published:** 1948

**Authors:** Charles Corfield


### The Bristol Medico-Chirurgical Journal

*" Scire est nescire, nisi id me
Scire alius sciret."*

WINTER, 1948.

### MEDICINE AND THE LAW

A summary of the Presidential Address that should have been delivered in October, 1939, at the opening of 1939–40 Session of the Bristol Medico-Chirurgical Society *

BY

Charles Corfield, M.D., Barrister-at-Law.

It seems rather a curious thing that of all the other arts and sciences, such as engineering, teaching, music, architecture and the church, that might occasionally be found to attract medical men who are looking for new worlds to conquer, the senior branch of the Law, that of the Bar, is the one which appeals to them most often. Perhaps it is because the practice of these two professions is concerned with the frailties and aberrations of human conduct that medical men sometimes find themselves attracted by the problems which concern the lawyer in his daily life.

The Bar finals take place four times annually. Scattered among the names of the successful you will find some with medical qualifications. Analysis of these would probably show that a large percentage are in government jobs, either at home or abroad, or perhaps in the Army or Navy, a qualification of the Bar being thought to help promotion in such cases. There remain over some who want to become coroners and lastly those who have decided to practise at the Bar for a living. One may say here in passing that whereas all graduates in medicine intend to practise it in some form, only a very small proportion of those who pass the Bar finals ever expect or try to earn their living at the Bar.

A word about the course of study for Bar students as compared

* Owing to the outbreak of war the session was cancelled.

with medicine. For the latter there are many opportunities to train and qualify both in London and the provinces. But to become a barrister in England there are only four institutions that can offer such facilities.

These are the four Inns of Court, all in London : Inner and Middle Temple, Lincoln's Inn and Gray's Inn. The general supervision of the curriculum is under the Council of Legal Education, a body something similar to the General Medical Council, without the judicial functions ; the latter being exercised by the benchers of each Inn.

The method of reading for the Bar is first, as in medicine, a general educational test. Then choose an Inn much in the same way that one would a college at Oxford or Cambridge. An application form is procured from the Inn of one's choice and this has to be signed by two barristers of not less than five years' standing. One is then admitted a student : naturally, after payment of fees. It is then fairly plane sailing. If not a member of a university, six dinners a term are eaten. There are four terms in the year, Hilary, Easter, Trinity and Michaelmas. As a member of a university, it is considered perhaps that one's table manners are likely to be a bit better and so only three dinners have to be taken. That is of course the minimum : one could dine every night if one so wished, and a very pleasant function it was till the bombing from 1940 onwards made these things impossible.

Curiously enough, dining and paying fees are the only conditions precedent for sitting for the examinations. Anybody, however, who wants to make a good show, should certainly attend the lectures laid down in the syllabus. They are given at the various Inns, under the auspices of the Council of Legal Education.

The subjects of the examinations are : (i) Roman Law, which is the foundation of most civilized codes ; (ii) Constitutional Law; (iii) Criminal Law; (iv) The Law of Evidence and Procedure, and (v) the final examinations consisting of Common Law and Equity with two special subjects, one of mine being Bankruptcy Law. I have had occasion since to consider it was very appropriate.

The student who merely wishes to qualify on the book, so to speak, need do no more than attend lectures. But those who wish to practise for a living will certainly go into the Courts which are open to them and see something first-hand of the practice of Law there. In addition, they will read in the Chambers of some busy junior at a cost of say a hundred guineas for six months. This is rather like the old apprentice system for medical students. It shows one the practical side of the work in a barrister's office, and it is a *sine qua non* for anyone wishing to practise. The curriculum thus outlined for qualifying at the Bar is obviously a very different proposition from that of graduation in Medicine. The whole period of study occupies three years of four terms a year.

As has been said, most medical men who get called to the Bar do it either because they are in government employment, or in the running for a coronership. But there have actually been some doctors who have entirely abandoned medicine and attained considerable success at the Bar. It might perhaps be of interest to give a short account of some of them.

Easily the most distinguished of these was the first Viscount Finlay. He was a medical student at Edinburgh, graduating at twenty-one. For two or three years he did resident appointments and locums in Edinburgh, and then he came to London to read for the Bar. He got called, and started practice on the Midland Circuit. In the course of time he built a sound common law practice and in a few years became M.P. for Inverness, Solicitor- and Attorney-General and subsequently Lord Chancellor, being, I think, the first medical man to hold that very high office. His son who died recently was a Lord Justice of Appeal.

Up till four years ago the Oxford County Court Circuit was presided over by Judge Cotes-Preedy. In his younger days he qualified at St. George's Hospital and was a House Physician there. He, like Lord Finlay, after doing some hospital appointments got called to the Bar and joined the Oxford Circuit. In a few years he developed a busy practice and eventually taking silk, became a leader at the Admiralty, Probate and Divorce Bar. He was made a County Court Judge in 1934, holding this office till his death in 1944.

Another interesting career is that of the present Senior Referee of the High Court of Justice—Sir Tom Eastham. He took the M.B., Ch.B. (Victoria) in 1902, and after doing some resident hospital work read for the Bar, getting called in 1904. He joined the Northern Circuit, and in course of time took silk, became a bencher of his Inn (Lincoln), Recorder of Oldham, and ultimately was given his present position.

One must consider these three in the sense of a complete breakaway from their first love. I do not think any of them was ever briefed in a case where medical knowledge was of any help or advantage, but in the case of Judge Cotes-Preedy I do not doubt that he found that this was quite useful and probably sometimes embarrassing to the medical witnesses.

Those who take part in Workmen's Compensation cases might know a monumental tome on this subject by Ruegg and Knocker. Douglas Knocker was a medical man, who qualified and after that practised for four or five years. He is the only man I know who for a while served two masters, so to speak. Having got called to the Bar and finding himself with small means, he ran a cheap cash practice near Liverpool Street Station, with very little visiting. He used to spend the early part of the morning there, then go down to his Chambers in the Temple, do his legal practice till four or five and then resume his medical duties once more. Later on his work at the Bar grew sufficiently for him to sell the other means of livelihood. This of course happened over thirty-five years ago. Under present conditions it is doubtful whether it could be carried through successfully.

Another one-time doctor was Ingleby Oddie. He was a naval surgeon, and, retiring on pension or gratuity, got called and practised at the Bar. He appeared as a junior in the celebrated Crippen case. Subsequently he was made a London coroner. A medical named G. C. Kingsbury was in practice a good number of years, and then got called to the Bar, and attached himself to the Central Criminal Court. I remember very well listening to him cross-examine the late Sir Victor Horsley who was giving evidence in some criminal case. His medical knowledge happened to be of great help in this particular instance, and I recall it as a very fascinating duel.

Another medical man had a less claim to fame. As he is still alive I prefer not to mention his name. He was a M.O.H. and got called to the Bar. Shortly after this he addressed a letter to the Town Clerks of the various London boroughs saying he was making a speciality of litigation connected with certain Public Health Acts. One of these circulars was sent to his Inn of Court, and, after being called before them, he was disbarred for two years. About five years ago the Senior Clerk of Arraigns at the Old Bailey died. In reading his obituary notice in *The Times* it was interesting to see that he had been a medical man in practice for nearly twenty years. At the age of forty-three he was called to the Bar, and was at the Central Criminal Court for about fifteen years, being then given this official post on the Old Bailey legal staff.

It is very probable that there are barristers practising at the present time who started life as medical men. I have sometimes been asked if I know of any lawyers becoming doctors, rather a different proposition perhaps, and certainly a much more strenuous one. I only know of two. In the famous " Brides in the Bath " case, an enterprising murderer used to insure his wives and then drown them. He did this three times successfully. In the second wife-murder case a Dr. French gave evidence. Although in the thirties, he had only been qualified for two years, having started his career as a solicitor.

I remember some ten years ago reading the obituary notice of a doctor who had recently died. It appeared that earlier in life he started at the Bar, getting a good junior practice on the S.E. Circuit. At the age of thirty he contracted tuberculosis and for some reason or other went to Australia. Out there he took up medicine, qualified at Melbourne, drifted back to this country and settled down as a country practitioner in the West of England, and a very good one at that.

As a rule practitioners of law and medicine pursue their paths in life perhaps in very different circumstances. But there are some problems dealing with human frailties common to them both, and there are other problems, social and economic, with which they are also equally concerned. Nearly forty years ago it occurred to a few enthusiastic lawyers and medical men that they might pool their experiences and discuss some of these common problems, so they started a society called the Medico-Legal Society. The terms of membership included not only doctors and lawyers, but anyone who was interested in the wider problems which concerned them both. It has grown from strength to strength and owing to its wide scope of membership includes a distinguished number of men in all walks of life. The president is elected annually and is often a High Court Judge. When one finds that people like Bernard Shaw and the late Bernard Spilsbury contributed frequently to the debates, it can be surmised that the standard of discussion was at a high level and often most entertaining. Some years ago when the medical societies of London united to form the Royal Society of Medicine, it was proposed that the Medico-Legal Society should come into the scheme, but the large lay membership proved an insuperable obstacle. Notwithstanding that it is probably true to say that no other scientific society has attained such standing. It is in touch with Government departments and has sometimes had referred to it legislative proposals in the rough, so to speak, for comment and suggestion.

I have alluded to the Medico-Legal Society at some length, because the scope of its activity shows that there are many subjects which doctors and lawyers can discuss with interest and advantage together. The present trend of social and health legislation makes it probable that this field will be greatly enlarged in the years to come.

